# The Association between Substance Abuse and Sexual Misconduct among Macau Youths

**DOI:** 10.3390/ijerph16091643

**Published:** 2019-05-11

**Authors:** T. Wing Lo, John W. L. Tse, Christopher H. K. Cheng, Gloria H. Y. Chan

**Affiliations:** 1 Department of Social and Behavioural Sciences, City University of Hong Kong, Hong Kong, China; john.tse.ss@cityu.edu.hk (J.W.L.T.); chris.cheng@cityu.edu.hk (C.H.K.C.); 2 School of Social Sciences, Caritas Institute of Higher Education, Hong Kong, China; chanhongyee2004@yahoo.com.hk

**Keywords:** peer influence, school attachment, substance abuse, sexual behavior, youth, Macau

## Abstract

This study investigates how peer influence, school attachment, and substance abuse are related to sexual behavior, with particular interest in exploring the relationship between substance abuse and sexual misconduct, while using a stratified random sample of adolescents in Macau. Mediation analyses were employed. The results show that substance abuse, apart from susceptibility to peer influence and school attachment/commitment, was significantly related to sexual misconduct. Substance abuse was the best predictor of sexual misconduct, and it significantly mediated the relationship between susceptibility to peer influence, as well as school attachment and sexual misconduct. This reflects that the use of substances, including drugs, alcohol, and cigarettes, can be viewed as a catalyst for triggering engagement in sexual misconduct. The implications of this study involve taking measures to reduce the rate of substance abuse as a way of decreasing sexual misconduct in adolescents. Future research directions in exploring the relationship between adolescent substance abuse and risky sexual behavior are discussed.

## 1. Introduction

Macau is a small city in southern China with a population of 0.6 million, Chinese and overseas capital has been investing in the gaming industry since the mid-2000s, turning this former Portuguese colony into an entertainment city [1]. Despite its rapid economic growth and social change, Macau is relatively underdeveloped in youth research [2,3,4]. Ample research evidence in the West has suggested that negative peer influence and poor school attachment affects the sexual behavior of young people [5,6,7,8], while substance abuse can also have an impact on adolescent sexual behavior [9]. Given that Macau is a mixture of Eastern and Western cultures, it is worthwhile examining whether similar findings will be observed among young people in the context of Macau. A study on the relations between school attachment and commitment, negative peer influence, substance abuse, and sexual misconducts among Macau youth would be most valuable.

Negative human activities, such as substance abuse and risky sexual behavior, which are common in the West, are also prevalent among adolescents in Macau [3]. In the West, research studies indicate that there is a strong relationship between substance abuse and risky sexual behavior among adolescents [10]. While risky sexual behavior focuses more on negative health aspects, such as sexually transmitted diseases, there is relatively little research attention regarding sexual misconduct of adolescents in Chinese communities. Sexual misconduct puts the emphasis on the legitimacy and appropriateness rather than the health consequences of sexual behavior. There has been very limited attention given to the study on adolescent sexual misconduct. Little is known regarding the relationship between substance abuse and sexual misconduct for adolescents under 18 years old in Macau. There is a need to fill the research gap and explore whether negative peer influence, poor school attachment, and substance abuse are factors that contribute to sexual misconduct. Since Macau is a sociocultural fusion of Chinese and Western countries, a study on the relationship between substance abuse and sexual misconduct may have wider implications for cities with fast growing economies and social changes. This helps shed light on policy formation.

The present study investigates the relationship between peer influence, school attachment, and substance abuse and sexual misconduct among Macau youths. Its objective is not to investigate the prevalence of substance abuse, but to examine whether substance abuse is related to sexual misconduct among youths in Macau.

### 1.1. Adolescent Sexual Misconduct

The situation of sexual misconduct among adolescents worldwide is rather bleak. A survey in the United States (U.S.) documented that 7.4% of students between Grade 9–Grade 12 reported having been physically forced to have sexual intercourse without consent [11]. The prevalence of this kind of sexual coercion was higher among females (11.3%) than males (3.5%). A survey in the U.S. on sexual assault (including rape, sodomy, and caressing) indicates that 37% of children under the age of 6 years, 42% of children between ages 6–11, and 33% of adolescents between ages 12–17 were sexually victimized by a juvenile offender under the age of 18 [12]. On the other side of the world, the prevalence of compensated dating (*Yuan Jiao*; an Eastern form of underage prostitution, literally means the “exchange of intimate sexual behavior with monetary rewards) in Hong Kong has been estimated to be 2.7%. These adolescents were more likely to exhibit risky sexual behavior and resulted in unintended pregnancy [13].

Sexual misconduct is a broad term that encompasses any inappropriate behavior of a sexual nature that is of lesser offense than sexual crime (such as rape), but is regarded as violating the social norms of society. Examples of sexual misconduct include, but are not limited to, unwelcome kissing or body touching, participating in erotic games, prostitution or compensated dating, sexual coercion, and so on. The definition of sexual misconduct also varies according to the age and mental capacity of the individuals, but it is not solely determined by the behavior per se. A behavior can be defined as sexual misconduct when it is committed by or to a person of underage or of under-functioning mental capacity (e.g., under the effect of alcohol or drugs), but the same behavior may not be a misconduct when performed by an adult who has obtained consent from another adult of normal mental functioning. For example, visiting a sex worker or participating in erotic games can be regarded as sexual misconduct when an adolescent under the age of eighteen performs it.

The laws of Macau (and China where Macau is its special administrative region) define young people under eighteen years old as a minor. Chinese parents have held a highly conservative attitude towards the sexual behavior of minor. Previous research on Chinese parents found that, while they recognized the changing social norms regarding sexuality, they were ambivalent regarding the sexual activities of unmarried children aged 18–24, and were largely opposed to pre-marital sexual activity [14]. Among Taiwanese school students, the fear of being judged, parental rejection, and pregnancy control their desire for premarital sex [15]. In mainland China, research found that the communication regarding adolescent romantic and sexual engagement experience between young people and their parents was consequence-based and prohibitive in nature, such as health risks, sociocultural risks, and detriments to education and future prospect, but it lacked useful romantic and sexual knowledge [16]. All in all, the research findings in these Eastern societies reflect the concerns about further loosening of the conventional sociocultural beliefs in romance, sexuality, family, and marriage.

### 1.2. Susceptibility to Peer Influences

Peers can have a substantial influence on adolescents. Studies have shown that it is innate for humans to mingle with like-minded individuals [17,18]. Like family, peer interaction serves as a crucial socializing agent during adolescence. Studies have shown that youths’ respective peer groups support their engagement in sexual abstinence and those who are sexually active. Being in groups of people who participate in early sex, youths might gradually perceive the practice as morally acceptable, especially in long-term relationships if precaution has been taken [19]. In effect, it has been supported by studies that a peer group exerts significant influences in affecting or enhancing decisions regarding sexual activities [7,8].

While affiliating with peers who engage in sexual misconduct or early sex can enhance such deviant behavior, it has been found that a greater sense of personal control over sexual behavior is one of the strongest factors in delaying sexual debut among youths [19,20]. Such resistance to conform to peer norms seems to be an effective protective factor against inappropriate sexual activities. Nevertheless, the similar concept of susceptibility to peer influence as a resilient agent has been minimally studied. Many studies that contribute to this concept are outdated [21,22,23], and one can question the validity of using such results to explain the present situation.

### 1.3. School Attachment

School attachment or connectedness can be perceived as the youth’s view on relationships with people at school, affiliation with the school, and approach toward the school’s importance [24]. It is the close emotional relationships with those at school and the commitment to strive for success in school that inhibits deviant behavior, such as sexual activity [25]. Sabia [26], provides several theoretical reasons as to why youths taking part in early sexual behavior might become less attached to school, which implies that early sexual behavior can further influence subsequent attachment to school. From teen pregnancies or sexually transmitted infections to psychological or physiological effects of early teen sex, interest, and concentration in school will gradually be lost, as engaging in sexual activities impedes their schooling.

Several theories highlight the different factors that link youth sexual behavior and school attachment. Early sexual behavior can affect decision making in schools, as highlighted by the problem behavior syndrome theory [27,28,29,30,31,32,33]. This theory predicts that problem behaviors, like early sexual experiences, trigger a change in an adolescent’s mindset to explore other anti-social behaviors, such as skipping school and taking part in delinquent behaviors that would lead to suspension from school. The economic theory of fertility implies that those who are suspended from school have low educational aspirations, feel secluded at school, and skip lessons are most likely to engage in sexual behavior at an early age [34].

Many cross-sectional and longitudinal studies have been conducted to provide empirical support for the association between adolescent sexual conduct and school connectedness. Studies have found that attachment to school can be a protective factor in adolescent sexual and reproductive health (ASRH) outcomes of ever having sex [35,36,37], early sexual debut [38,39], and frequency of sex [40,41]. Furthermore, Sabia [26] found that there is a negative relationship between school attachment and adolescent sexual misconduct. Early teen sex is found to be associated with a higher probability of unexplained absences and even suspension from school, weakened school attraction, and low aspirations to attending college. Madkour, Farhat, and Halpern [42] also reported that school attachment is inversely related to early sexual experience between both genders, and Carter et al. [5] identified school engagement as a protective factor in predicting risky sexual behavior.

Basile and colleagues [43] reported that youths identified as sexual violence perpetrators had lower school belongingness at earlier time points in middle school when compared to non-perpetrators, albeit reaching similar levels in high school. Other studies that were conducted in the US have found that youths who drop out of school tend to increase their sexual behavior. They are more likely to initiate sex earlier, fail in using contraception, become pregnant, and give birth [44,45,46,47]. All in all, many studies have illustrated that youths who have lower school attachment are more likely to engage in sexually inappropriate behavior.

### 1.4. Substance Abuse

Interestingly, literature regarding the relationship between substance abuse and risky sexual behavior can indirectly explain the relationship between substance abuse and sexual misconduct. Researchers claimed that substance abuse leads to risky sexual behavior due to the disinhibition effect [10,48]. In other words, adolescents who abuse a substance are less likely to exercise self-restraint and exhibit sexual misconduct or inappropriate sexual advances. On the other hand, other researchers [e.g., 49] took the opposite stance and claimed that risky sexual behavior leads to substance abuse. The plan to partake in sexual behavior or sexual misconduct may cause one to abuse a substance [49]. Therefore, instead of regarding substance abuse as having a disinhibition effect, substances are used in a deliberate manner and they serve as a way of engaging in sexual misconduct.

Empirically, youth deviant behavior, such as substance abuse, has been shown to be associated with having liberal sexual opinions or being sexually active [33,50]. Prior studies have shown that the role of substance abuse positively correlates with engagement in risky sexual behavior among youths [9,49,51,52,53,54,55]. Anderson and Mueller [56] reinforced these findings, although a strong association exists relating risky sex with illicit drug use and not with alcohol use. The effects of illicit drugs and alcohol can be used as an approach to pave the way for an intimate experience with a new partner or to enhance the level of sexual pleasure [57]. However, experimentation with substances during adolescence might be an unintentional catalyst for sexual behavior [58], and both of the variables can be seen as part of a thrill-seeking personality trait [59]. Grossman et al. [57] also asserted that substance abuse has many negative impacts, because it can hinder personal decision making and judgment, resulting in multiple sexual partners, failure to use any contraceptive methods, and communication impairments that affect personal relationships. According to alcohol myopia theory [60], alcohol influences one’s information processing, which causes the continuous processing of immediate, instigating cues (e.g., arousal), while also suppressing the processing of distal and complex ones (e.g., concerns about pregnancy). These findings show that, due to the disinhibition effect of substance abuse on complicated information processing, the use of substances increases sexual misconduct.

Other research [61] suggests that the relationship between sexual behavior and substance abuse is not absolute, but it might involve other intervening variables. For example, a reason for substance abuse when choosing to engage in inappropriate sex might be due to one’s attempt to cope with society’s pessimistic view of sexual behavior [61]. Cooper, Peirce, and Huselid [62] further argued, “if the acute effects of alcohol cause one to take more risks, then drinking and risk behaviors should be temporally linked” (p. 251). This adds uncertainty to the relationship between substance abuse and inappropriate sex.

### 1.5. The Present Study

None of the aforementioned literature is related to youth sexual misconduct in Macau. Macau, dubbed the “Las Vegas of the East”, which has a unique mixture of Chinese and Western sociocultural characteristics, deserves a study of its own. Against this backdrop, the present study aims to investigate the impact of substance abuse on the sexual behavior of adolescents in Macau. The studies that are reviewed above have demonstrated that taking drugs, drinking, smoking, school attachment, and susceptibility to peer pressure can have a significant impact on the rise of sexual behavior. Although these factors on sexual behavior have been discussed in Western research, the relative intensity of these factors in predicting sexual behavior among youths living in a Chinese casino city is unclear. Although personal control, resistance against peer pressure, and attachment to school serve as protective factors against risky sexual behavior [19,20,25], the use of substances (e.g., alcohol) can cause a disinhibiting effect that loosens the protective barrier against risky sex. Additionally, there are studies showing inconsistent effects of substances (e.g., alcohol) among different age groups [63]. The present study collected data from a large sample of Macau youths in 2014 [64] to fill the gaps regarding youth substance abuse. We hypothesise that substance abuse might be the crucial factor triggering sexual behavior among youths in Macau by focusing on the possible mediating effect of substance abuse (taking drugs, drinking, and smoking) in the intrapersonal and interpersonal domains.

## 2. Methods and Analysis

### 2.1. Predictor Measures

The variables of school attachment, susceptibility to negative influence from peers, taking drugs, drinking, and smoking were assessed in the present study for examining the effect of intrapersonal and interpersonal variables on engagement in sexual behavior.

#### 2.1.1. Substance Abuse

Substance Abuse includes three items: drug taking, smoking, and alcohol drinking. According to Hong Kong laws, these three acts are illegal when committed by people under the age of eighteen. The format of the questions was adopted from the Self-Reported Delinquency scale of Baldry and Farrington [65]. The respondents were asked to indicate whether they ever committed these acts in the last three months on a five-point Likert scale, ranging from 0 (never), 1 (rarely), 2 (sometimes), 3 (often), to 4 (always). This scale had acceptable internal consistency reliability (Cronbach’s α = 0.69), mean = 1.03, SD = 1.97.

#### 2.1.2. Attachment and Commitment to School

The scale on school attachment and commitment was adopted from the Simcha–Fagan and Schwartz scale [66], which was designed for measuring the extent of the adolescent’s attachment and commitment to school. The scale consisted of thirteen statements, such as “I love my school” and consisted of a four-point Likert scale, from 1 (absolutely disagree) to 4 (absolutely agree). A higher score represented strong attachment to school. The scale had good internal consistency reliability (Cronbach’s α = 0.77), mean = 2.95, SD = 0.39.

#### 2.1.3. Susceptibility to Negative Influence from Peers

Three items, from Dalton [67] and Dielman et al. [22], measured susceptibility to negative influence from peers. Examples of questions included statements, such as “If your friends ask you to watch a movie together when you have to prepare for quizzes, would you go with them?” This measure used a four-point Likert scale, and the response options were (1) surely no, (2) no, (3) yes, and (4) surely yes. The scale had acceptable reliability (Cronbach’s α = 0.70), mean = 1.72, SD = 0.58.

### 2.2. Outcome Measure

#### Sexual Misconduct

With reference to the social norms, in particular, the Chinese sociocultural values in relating to sexual activities, a team comprising of four professional social workers and psychologists identified behaviors that were deemed to be sexually inappropriate for adolescents that were aged 17 or below. After deliberation, the team identified four behavioral indicators that were regarded as violation of traditional sex norms among adolescents in Chinese society. The items were: In the last three months, I… “had sexual intercourse with an opposite sex”; “engaged in sexual activities for commercial purposes (such as prostitution or compensated dating)”; “participated in erotic games”; and, “forced a person to have sex”. The respondents were to report the frequency of these behaviors on a five-point Likert scale, with the options including (0) never, (1) seldom, (2) sometimes, (3) frequently, and (4) always. This scale had rather high internal consistency reliability (Cronbach’s α = 0.87), mean = 0.40, SD = 1.65.

### 2.3. Sampling and Procedure

The present paper reports a small part of a much larger study that was commissioned by the Macau government, with a sample including both school youth and their parents. After ethical review was approved by City University of Hong Kong (9231042), data collection was carried out with assistance being provided by the Social Work Bureau of the Macau Government. The target group was full-time secondary school students (and their parents) who were selected by a multistage stratified sampling approach. We first divided the city into eight geographic clusters and then randomly selected two to three secondary schools from all schools in each cluster (accounting for the student population size in each cluster) to ensure the representativeness of the selected samples. Among the sampled schools, stratified random sampling was used to select the classes of participants, giving an even distribution of students from each form. Subsequently, we sought the consent of the sampled schools to participate in the study. Through the consented schools, we invited students and their parents to participate in the study. Two research assistants for those respondents who gave consent administered data collection. The two assistants grouped the consented students in a school classroom to fill in the questionnaire, while the parents were to separately fill out their questionnaires at home. In view of the potential limitations of self-report, such as self-enhancement bias or social desirability responding, school teachers were not present in the venue and the respondents were ensured of anonymity and confidentiality before the data collection was started. When the students were filling in the questionnaires, they were not allowed to interact with each other. When they completed, they put the questionnaires in a sealed collection box that was prepared by the assistants and they were directly dispatched to the research team without passing through the hands of a third party. The procedure was to ensure the confidentiality of data and the anonymity of the respondents. Ultimately, 2755 students from 12 secondary schools participated in the study. Since the present paper investigated the sexual misconduct of minors, the findings of the parents and adult students (aged 18 or above) were not included in the analysis. In the present paper, a sample of 2555 students in F.1 (grade 7) to F.6 (grade 12) participated in the study; they were aged from 11–17 years of age.

### 2.4. Analytic Strategy

**Figure 1:**
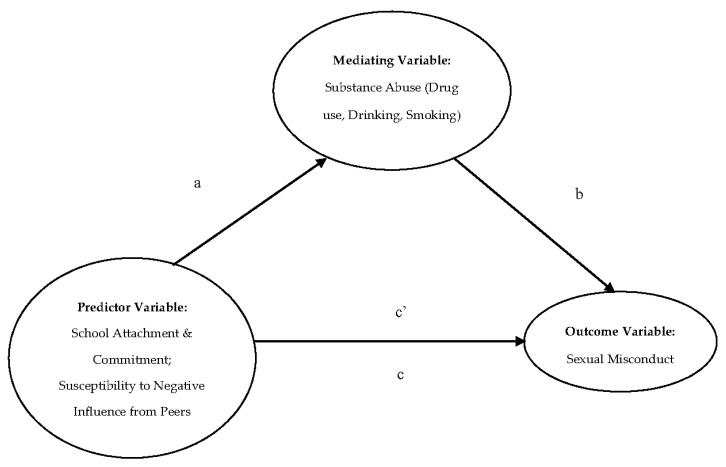
Conceptual model of the mediating regression analysis.

Statistical analyses were conducted while using IBM SPSS for Windows 24.0 (SPSS Inc., Chicago, IL, USA). Descriptive statistics, reliability tests, and Pearson’s bivariate correlations were first examined. Hierarchical regression analyses were conducted to test the predictive effects of the predictor variables on the outcome variable (sexual misconduct). Demographic variables (age, gender) were entered in the first step as the controls. Predictor variables (commitment/attachment to school, susceptibility to negative influence from peers, substance use) were entered in subsequent steps to check for the variance that was explained by the regression models. The predictive effects of various predictors were indicated by the standardised beta coefficients. The potential problem of multicollinearity was checked by the variance inflation factor (VIF) index. To analyse the mediating effects, the widely used principle of Baron and Kenny [68] was employed. Figure 1 shows the conceptual model of the mediating analysis, where the total effect and direct effect of the IV are expressed by c and c’, respectively, and the indirect effect of IV on DV through the mediating variable (MV) is indicated by the path a × b. The Sobel test and the confidence intervals under bootstrapping analysis tested the statistical significance of the mediating effect. The Process macro that was developed by Hayes [69] was adopted for the mediation analysis.

## 3. Results

### 3.1. Sample Characteristics

The sample was composed of 2555 adolescents (53.3% female) that were aged between 11–17 years (M = 14.84, SD = 1.57) from F.1 (grade 7) to F.6 (grade 12) levels of study. With respect to their parental background, the majority had attained junior secondary level (father: 28.5%; mother: 35.0%), less than a quarter were matriculated (father: 24.8%; mother: 22.8%), approximately one-fifth of them had received primary level education (father: 19.3%; mother: 18.8%), and about 10% of them had attained tertiary level education (father: 11.6%; mother: 10.0%). In relation to the employment status of the parents, the majority of fathers worked as semi-skilled workers (29.6%) and more than half of mothers worked as non-skilled workers (57.5%). The second distinguished type of job is also different between genders, with 19.4% of fathers working as skilled workers and 12.4% of mothers working as semi-skilled workers. With regard to household income (1 USD ≈ 8 MOP), roughly one-quarter of the families had a monthly household income below 15,000 MOP, 18% of the families had a monthly income of 15,000–24,999 MOP, and 13.8% earned 25,000 MOP or more per month. Table 1 presents a more detailed description of the sample characteristics.

### 3.2. Descriptive Statistics

Table 2 showed the descriptive statistics and reliability coefficients of all measurement scales. All of the measurement scales had acceptable internal consistency reliability with Cronbach’s α ranging from 0.69–0.87. The summary statistics showed that the level of school attachment and commitment was about the medium level (Mean = 2.95, SD = 0.39). The negative influence from peers was quite common among the adolescent respondents (Mean = 1.72, SD = 0.58). While sexual misconduct (Mean = 0.40, SD = 1.65) and substance abuse (Mean = 1.03, SD = 1.97) were not very common among the respondents, some extent of these acts was still reported.

### 3.3. Regression Analysis on Sexual Behavior and Mediation Analysis

Hierarchical regression analysis was conducted, such that sexual misconduct was specified as the outcome variable, while school commitment/attachment and susceptibility to negative influence from peers were the predictor variables, followed by substance use (hypothesised as the mediating variable); age and gender were controlled in the first step (see Table 3). The potential problem of multicollinearity was checked, and it was confirmed that the VIF values were low (ranging from 1.249–2.035), which indicated that there was no problem of multicollinearity. The results showed that the hypothesised models were statistically significant. School commitment and negative peers influence accounted for 11% of the variance in sexual misconduct (*R^2^* = 0.11, *F*(4, 2837) = 87.32, *p* < 0.001), and substance use added another 9.6% in predicting sexual misconduct (*R^2^* = 0.206, *F*(5, 2836) = 146.76, *p* < 0.001). Among the predictor variables, substance use had the largest effect on sexual misconduct (*β* = 0.396, *p* < 0.001). Although the effects of negative influence from peers and school attachment/commitment were statistically significant (*β* = 0.27 and −0.07, respectively, *p* ≤ 0.001), their effects on sexual misconduct drastically dropped when substance abuse was controlled (*β* = 0.047 and −0.036, *p* = 0.043 and 0.065, respectively). This suggests that substance abuse had the strongest predictive effect and it possibly mediated the effects of peer influence and school commitment on sexual misconduct. Further mediating analyses were conducted to delineate the mediating role of substance use.

**Figure 2:**
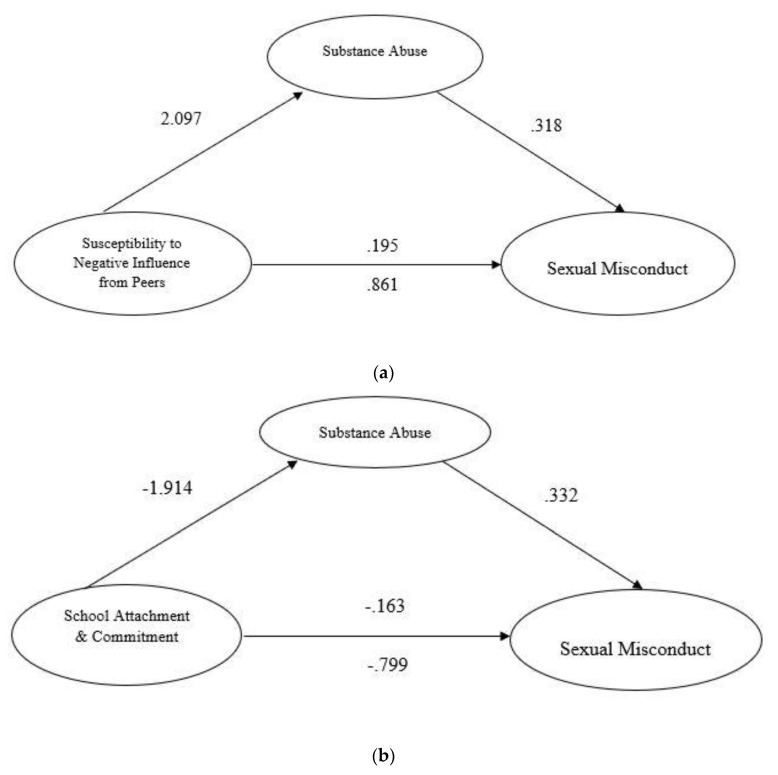
(**a**) Mediating model of substance abuse on the relationship between peer influence and sexual misconduct. (**b**) Mediating model of substance abuse on the relationship between school attachment and commitment and sexual misconduct.

Simple mediation analyses (model 4 in Hayes [69]) were conducted, in which the same variables as above were input as predictors (school attachment/commitment, susceptibility to negative influence from peers), mediator (substance use), and outcome variable (sexual misconduct). Both the Sobel and bootstrapping tests were consulted to indicate the significance of the indirect effect of the predictors under the mediation of substance abuse. The results showed that both of the predictor variables (school attachment, peer influence) had significant total effects (direct and indirect) on sexual misconduct, but their effects were significantly reduced when substance use was controlled. On the other hand, substance use had the strongest direct effect on sexual misconduct, even when the predictors (school attachment, peer influence) were controlled, and it significantly mediated the effects of both the predictors (school attachment, peer influence) on sexual misconduct. The Sobel test and bootstrapping statistics showed that the indirect effect of school attachment via substance use (i.e., path axb) was significant (Coefficient = −0.64, *Sobel Z* = −16.49, *p* < 0.0001). The same was found for negative peer influence—that is, the indirect effect of negative peer influence via substance abuse (path axb) was also significant (Coefficient = 0.67, *Sobel Z* = 17.17, *p* < 0.0001). Lastly, the reverse procedure (i.e., reversing the predictor and the mediator in the mediation models) was carried out to check whether school attachment and/or negative influence from peers might have a mediating role in explaining the effect of substance abuse on sexual misconduct. The results confirmed that substance abuse had the strongest direct effect and mediating effect, and it was not mediated by the school and peer factors. Figure 2a,b show the mediating role of substance abuse in the relationship.

## 4. Discussion

The results of the present study showed that susceptibility to peer influence, drug-taking, drinking, smoking, and school attachment are related to sexual misconduct in adolescents under 18 years old. Among all of the factors affecting adolescent sexual misconduct, substance abuse (including drugs, alcohol, and cigarettes) has the largest predictive power. Additionally, substance abuse displayed a significant mediating effect in the relationship between susceptibility to negative influence from peers as well as school attachment and sexual behavior. The results reflect that substance abuse is a significant crucial factor that triggers sexual misconduct among adolescents in the Macau context. It is important to note that the present results are consistent with ample research findings, suggesting a positive association between substance abuse and risky sexual behavior [10,70]. 

The present study lends support to the issue of directionality of substance abuse and sexual behavior. Some of the researchers asserted that substance abuse leads to risky sexual behavior [48,70], while others took the opposite stance and claimed that risky sexual behavior leads to substance abuse. Recent research confirms that early sex (at age 15 or younger) is predictive of substance abuse in adulthood [71]. The intention to participate in sexual behavior (e.g., on a date) might cause one to abuse a substance [49], or it provides an excuse for sexual advances that might be considered to be inappropriate. In other words, instead of regarding substance abuse as having a disinhibition effect, substances are used in a planned manner and they serve as an excuse for engaging in sexual behavior. Cooper [49] claims, “(T)he intention or desire to have sex may precede and cause drinking, rather than the reverse” (p. 20).

The evidence in the present study confirms that susceptibility to negative peer influence, low school attachment and commitment, and substance abuse are significantly predictive of sexual misconduct. This is consistent with Western findings that substance abuse increases sexual activities [33,50], particularly risky ones [e.g.,9,49,51]. It is also in line with Western findings that peer group influences affect youths’ decisions regarding sexual activities [7,8,72], and that non-attachment to school has impact on sexual misconduct [35,36,37]. However, a major discovery of the present study, which has not been reported in the Western studies, is that, as shown in our mediation analyses, substance abuse is a significant partial mediator that contributes to the association between peer influence, as well as school attachment and sexual misconduct. This reflects that substance abuse not only acts as a catalyst that triggers one’s sexual activity [33,50] and decreases one’s ability in executing decision making and judgment to control sexual behavior [57], but it also strengthens the effects of negative peer influence and low school attachment on sexual misconduct.

It is pertinent to carry out preventive intervention programmes in reducing rates of substance abuse at an early age, which, in turn, can reduce sexual misconduct among adolescents, since adolescent sexual misconduct is unlawful or unsanctioned behavior, and the present results confirm that substance abuse can predict sexual misconduct. Due to the legal and health consequences of substance abuse, there is an urgent need to enable adolescents to acquire the sufficient skills and awareness to reject inherently gratifying materials. One implication is to break a behavioral chain at an early stage that is to intervene before substance abuse turns into a stubborn habit. There would be a delay in the onset of substance abuse among early adolescents if effective prevention programmes were implemented, resulting in a reduction of subsequent harms later in life. Lynskey [73] estimated that between 8% to around 12% fewer adolescents would have refrained from binge drinking, smoking regularly, and using marijuana. Though it is difficult to achieve long-lasting results in preventive work in schools, community interventions can be effective—for example, an increase the price of tobacco products, conducting anti-smoking campaigns in the media (including social media), and enforcing legislation ensuring that adolescents have difficulty purchasing tobacco products [74]. On the other hand, the screening of and treatment for adolescents regarding substance abuse by primary care physicians can also be useful [75].

Since the present results suggest that many adolescents below 18 years old engage in sexual misconduct, a practical implication is to promote better sexual health and minimize the possible negative impacts by offering sex education before one’s first sexual intercourse. According to the Macau Youth Research Association [76], sex education in Macau is insufficient and conservative. Sex education in Chinese societies is predominantly based on an abstinence-only approach [77]. The abstinence-only approach to sex education is theoretically sound, but such programmes often fail in practice. For instance, Shepherd, Sly, and Girard [78] compared the effectiveness of comprehensive sexuality and abstinence-only education programmes for students that were aged between 12–14 years. The results found that adolescents in the abstinence-only education were more likely to engage in unprotected sex. The effectiveness of group-based comprehensive sex education programmes implies that there is an urgent need to carry out such programmes to tackle adolescent sexual misconduct [79].

Although research on the bidirectional or reciprocal relationship between substance abuse and risky sexual behavior have been carried out [80,81], the process and mechanisms of substance abuse on adolescent sexual misconduct are not clear. Future research should examine the complex relationship between substance abuse and sexual misconduct. It is crucial to explore the bidirectional relationship between these important constructs. A longitudinal design with a focus on examining the causal relationship between substance abuse and sexual misconduct, while also taking important variables, such as different types of substance abuse, violence, sexual orientation, sexting behavior, and the process of decision making, could shed more light on the controversy.

## 5. Conclusions

As stated by the Government Information Bureau [82], youths in Macau are susceptible to peer influence in drug taking. This reflects that interpersonal factors could also influence Macau youths’ sexual misconduct. Western studies indicate that substance abuse is associated with other factors, including inadequate decision making, low impulse control, and avoidance of society’s negative view of sexual behavior [62,83]. The results of the present study show that substance abuse is significantly linked with sexual misconduct. Additionally, such substance abuse is partly instigated by susceptibility to peer influence and school attachment. Based on these results, the implication is that, if the objective is to promote adolescent health and minimize sexual misconduct, then it is pertinent that substance abuse preventive intervention is carried out systematically and sex education programmes are implemented in secondary schools as a form of accompanying intervention. In recent years, the Social Work Bureau of Macau [64] has funded NGOs to run several Community Youth Work Teams in different districts to work with youth at risk of substance abuse. The Bureau has also financed the Integrated Youth and Family Service Centres that provide service for families and children and to students in schools. The results of the present study suggest that the Bureau’s investment in these two youth services is in the right direction and is addressing a pressing problem that is faced by the Macau youths.

Nevertheless, a major weakness of the present study may lie in the measurement of sexual misconduct. Despite the statistically high reliability coefficient of the sexual misconduct scale, its content validity is open for discussion. In general, sexual misconduct can be regarded as any sexual behavior that is unsanctioned and deemed to be inappropriate the social norms. Among the four items measuring sexual misconduct, the item regarding sexual intercourse by adolescents may not be considered to be a sexual misconduct in some cultures, due to the fact that early sexual initiation is rather common amongst adolescents in some countries. For example, various reports in the United States found that about forty to more than fifty percent of American youths aged 15–17 had sexual intercourse [57]. On the contrary, only around 6–7% of the same aged adolescents in Hong Kong have sexual intercourse experience, and the mean age of first coital experience among youths is 19 years of age [84]. There is no official statistics regarding adolescent sexuality in Macau, but we believe that the situation should be similar to that of Hong Kong. When considering the fact that early sexual initiation has been widely recognized as an important social and health issue (e.g., [13,49,85]), and that coital experience among adolescents below the age of eighteen is violating the sexual norms in Chinese societies [14], we have included sexual intercourse in the measurement of sexual conduct. Further research may be needed to strengthen the construct validity of sexual misconduct.

The cross-sectional design is another limitation of this study. We are able to confirm that adolescent substance abuse plays a crucial role in explaining their sexual misconduct. However, readers must be cautious in interpreting this relationship as a causal one. Given the complexity of motivation for substance abuse and sexual misconduct, the current research was insufficient in specifying the causal direction between sexual misconduct and substance abuse, but our findings have confirmed the role of substance abuse. A longitudinal research design is needed to uncover the causal relationship. Moreover, there is a heavy reliance on recall of events and behavior by filling out a self-report questionnaire. Although a self-report method has the advantage over direct observation or private interview, especially for sensitive topics, such as sexual experience [86], the self-report strategy has the inherent limitation of tendency of self-enhancement bias or social desirability responding [87]. In the future, researchers may explore the use of mobile phone apps or social networking sites to collect more immediate information regarding the participants’ feeling and experiences, especially about behaviors that are sensitive, such as sexual experience, drug taking, or alcoholic consumption.

To conclude, the study confirms the significant role of substance abuse (including drug, smoking, and drinking) of young adolescents in explaining the relationship between negative peer influence, low school commitment and sexual misconduct. Substance abuse has both the direct and indirect effects on sexual misconduct among young adolescents in Macau.

## Figures and Tables

**Table 1:** Sample characteristics (*n* = 2555).

Variable	%
Gender	
Male	46.7
Female	53.3
Age; M (SD)	14.84 (1.57)
11	1.0
12	9.3
13	13.4
14	17.7
15	20.9
16	20.4
17	18.2
Education	
Junior (forms 1–3)	66.1
Senior (forms 4–6)	33.9
Monthly Household Income (USD $1 = MOP $8)	
Below MOP $15,000	23.2
MOP $15,000–24,999	18.0
MOP $25,000 or above	13.8
Do not know	45.0
Father’s Education Level	
Primary or below	19.3
Junior Secondary	28.5
Senior Secondary	24.8
Tertiary	11.6
Do not know	15.8
Mother’s Education Level	
Primary or below	18.8
Junior Secondary	35.0
Senior Secondary	22.8
Tertiary	10.0
Do not know	13.4
Parents’ Marital status	
Married	79.1
Cohabiting	6.2
Separated or divorced	11.5
Widowed	2.6
Do not want to say	0.6

Note: M = Mean, SD = standard deviation.

**Table 2:** Descriptive statistics, internal consistency reliability, and inter-variable correlations of measurement scales.

	Mean	SD	No. of Items	Cronbach’s α
Sch Att	2.95	0.39	13	0.77
Peer Sus	1.72	0.58	3	0.70
Sexual	0.40	1.65	4	0.87
Subs	1.03	1.97	3	0.69

Notes: Sch Att = School attachment and commitment; Peer Sus = Susceptibility to negative influence from peers; Sexual = Sexual misconduct; Subs = Substance Abuse.

**Table 3:** Multiple regression analysis of sexual behavior predicted by school attachment, peer influence, and substance use.

	(Model 1)			(Model 2)			(Model 3)		
Predictor Variables	*β*	*SE*	*t*	*β*	*SE*	*T*	*β*	*SE*	*t*
Gender	−0.13	0.055	−6.94 ***	−0.07	0.054	−3.84 ***	−0.06	0.051	−3.54 ***
Age	0.046	0.014	2.49 *	0.012	0.013	0.685 *ns*	−0.023	0.012	−1.37 *ns*
Peer Sus				0.266	0.054	12.6 ***	0.047	0.059	2.02 *
School Att				−0.07	0.078	−3.39 **	−0.036	0.074	−1.84 *ns*
Subs							0.396	0.016	18.51 ***
(Model Statistics)									
*R*	0.14			0.33			0.45		
*R* ^2^ *change*	0.02			0.09			0.096		
*F*	28.94 ***			87.32 ***			146.75 ***		

Notes: Outcome variable = Sexual misconduct. Sch Att = School attachment and commitment; Peer Sus = Susceptibility to negative influence from peers; Subs = Substance Abuse. * *p* < 0.05, ** *p* < 0.01; *** *p* < 0.001.
